# Supplementary material for: Outcomes of a social media campaign to promote COVID-19 vaccination in Nigeria
Source: PLoS One. 2023 Sep 15;18(9):e0290757. doi: 10.1371/journal.pone.0290757 (PMC10503765; doi:10.1371/journal.pone.0290757)
Supplement: S1 File — (DOCX) [file pone.0290757.s001.docx]

Supplemental Table 1. Geographic Composition of the Baseline Sample, by Type of State of Residence (Treatment versus Comparison)

|  | Treatment State Participants (n=1100) | | Comparison State Participants (n=833) | | All Participants (n=1933) | |
| --- | --- | --- | --- | --- | --- | --- |
|  | n | (%) | n | (%) | n | (%) |
| Treatment States |  |  |  |  |  |  |
| Lagos | 987 | (89.7) | 0 | (0.0) | 987 | (51.1) |
| Anambra | 40 | (3.6) | 0 | (0.0) | 40 | (2.1) |
| Rivers | 32 | (2.9) | 0 | (0.0) | 32 | (1.7) |
| Niger | 17 | (1.6) | 0 | (0.0) | 17 | (0.9) |
| Bauchi | 13 | (1.2) | 0 | (0.0) | 13 | (0.7) |
| Sokoto | 11 | (1.0) | 0 | (0.0) | 11 | (0.6) |
| Comparison States |  |  |  |  |  |  |
| Plateau | 0 | (0.0) | 178 | (21.4) | 178 | (9.2) |
| Oyo | 0 | (0.0) | 77 | (9.2) | 77 | (4.0) |
| Federal Capital Territory | 0 | (0.0) | 60 | (7.2) | 60 | (3.1) |
| Kano | 0 | (0.0) | 53 | (6.4) | 53 | (2.7) |
| Kaduna | 0 | (0.0) | 43 | (5.2) | 43 | (2.2) |
| Enugu | 0 | (0.0) | 42 | (5.0) | 42 | (2.2) |
| Abia | 0 | (0.0) | 40 | (4.8) | 40 | (2.1) |
| Delta | 0 | (0.0) | 34 | (4.1) | 34 | (1.8) |
| Benue | 0 | (0.0) | 33 | (4.0) | 33 | (1.7) |
| Edo | 0 | (0.0) | 29 | (3.5) | 29 | (1.5) |
| Imo | 0 | (0.0) | 24 | (2.9) | 24 | (1.2) |
| Kwara | 0 | (0.0) | 22 | (2.6) | 22 | (1.1) |
| Borno | 0 | (0.0) | 19 | (2.3) | 19 | (1.0) |
| Akwa Ibom | 0 | (0.0) | 18 | (2.2) | 18 | (0.9) |
| Kogi | 0 | (0.0) | 18 | (2.2) | 18 | (0.9) |
| Ogun | 0 | (0.0) | 18 | (2.2) | 18 | (0.9) |
| Adamawa | 0 | (0.0) | 17 | (2.0) | 17 | (0.9) |
| Cross River | 0 | (0.0) | 17 | (2.0) | 17 | (0.9) |
| Ebonyi | 0 | (0.0) | 13 | (1.6) | 13 | (0.7) |
| Osun | 0 | (0.0) | 13 | (1.6) | 13 | (0.7) |
| Ondo | 0 | (0.0) | 11 | (1.3) | 11 | (0.6) |
| Gombe | 0 | (0.0) | 9 | (1.1) | 9 | (0.5) |
| Nasarawa | 0 | (0.0) | 7 | (0.8) | 7 | (0.4) |
| Yobe | 0 | (0.0) | 7 | (0.8) | 7 | (0.4) |
| Zamfara | 0 | (0.0) | 6 | (0.7) | 6 | (0.3) |
| Katsina | 0 | (0.0) | 6 | (0.7) | 6 | (0.3) |
| Bayelsa | 0 | (0.0) | 5 | (0.6) | 5 | (0.3) |
| Ekiti | 0 | (0.0) | 5 | (0.6) | 5 | (0.3) |
| Kebbi | 0 | (0.0) | 5 | (0.6) | 5 | (0.3) |
| Jigawa | 0 | (0.0) | 2 | (0.2) | 2 | (0.1) |
| Taraba | 0 | (0.0) | 2 | (0.2) | 2 | (0.1) |

Supplemental Table 2a. Item-Level Averages for the Five Cs of Vaccine Hesitancy, and Crude and Adjusted Differences at First and Second Follow-Ups

|  | Confidence | | Complacency | | Inconvenience | | Calculation | | Lack of Collective Responsibility | |
| --- | --- | --- | --- | --- | --- | --- | --- | --- | --- | --- |
| Levels (Mean and (SD)) | T | C | T | C | T | C | T | C | T | C |
| Baseline (n=1933) | 3.05 (0.94) | 2.97 (0.83) | 2.57 (0.95) | 2.69 (0.92) | 2.88 (1.08) | 2.82 (1.07) | 3.43 (1.07) | 3.54 (1.13) | 2.50 (1.00) | 2.51 (1.02) |
| First Follow-Up (n=1155) | 3.16 (1.03) | 3.13 (1.00) | 2.55 (1.01) | 2.58 (1.03) | 2.82 (1.08) | 2.69 (1.09) | 3.49 (1.06) | 3.55 (1.15) | 2.52 (1.04) | 2.47 (1.06) |
| Second Follow-Up (n=462) | 3.26 (1.16) | 3.27 (1.04) | 2.51 (1.06) | 2.50 (0.98) | 2.75 (1.11) | 2.73 (1.06) | 3.48 (1.10) | 3.47 (1.09) | 2.48 (1.05) | 2.35 (1.08) |
|  |  |  |  |  |  |  |  |  |  |  |
| Differences | Estimate | (p-value) | Estimate | (p-value) | Estimate | (p-value) | Estimate | (p-value) | Estimate | (p-value) |
| Crude at First Follow-Up | 0.03 | (0.591) | -0.03 | (0.515) | 0.13 | (0.044) | -0.06 | (0.198) | 0.05 | (0.328) |
| Adjusted at First Follow-Up | 0.04 | (0.487) | 0.01 | (0.837) | 0.04 | (0.524) | -0.02 | (0.684) | 0.01 | (0.815) |
| Crude at Second Follow-Up | -0.02 | (0.870) | 0.01 | (0.908) | 0.01 | (0.866) | 0.01 | (0.935) | 0.13 | (0.055) |
| Adjusted at Second Follow-up | -0.04 | (0.618) | 0.03 | (0.693) | -0.12 | (0.184) | 0.06 | (0.516) | -0.00 | (0.959) |

Supplemental Table 2b. Item-Level Averages for Pro-Vaccination Social Norms Indicators, and Crude and Adjusted Differences at First and Second Follow-Ups

|  | Friends | | Family | | People Close to You | | Nigerians | | Health Workers | |
| --- | --- | --- | --- | --- | --- | --- | --- | --- | --- | --- |
| Levels (Mean and (SD)) | T | C | T | C | T | C | T | C | T | C |
| Baseline (n=1933) | 3.11 (1.03) | 3.00 (1.05) | 3.16 (1.02) | 3.00 (1.00) | 2.51 (1.24) | 2.20 (1.63) | 2.76 (1.04) | 2.62 (1.01) | 3.36 (1.18) | 3.41 (1.17) |
| First Follow-Up (n=1155) | 3.16 (1.08) | 3.19 (1.03) | 3.17 (1.01) | 3.20 (1.05) | 2.63 (1.25) | 2.43 (1.52) | 2.80 (0.95) | 2.84 (0.99) | 3.50 (1.12) | 3.50 (1.11) |
| Second Follow-Up (n=462) | 3.15 (1.11) | 3.22 (1.08) | 3.26 (1.11) | 3.17 (1.09) | 2.67 (1.30) | 2.62 (1.50) | 2.86 (1.08) | 2.87 (0.99) | 3.52 (1.17) | 3.52 (1.08) |
|  |  |  |  |  |  |  |  |  |  |  |
| Differences | Estimate | (p-value) | Estimate | (p-value) | Estimate | (p-value) | Estimate | (p-value) | Estimate | (p-value) |
| Crude at First Follow-Up | -0.03 | (0.723) | -0.03 | (0.614) | 0.20 | (0.018) | -0.04 | (0.603) | -0.08 | (0.312) |
| Adjusted at First Follow-Up | -0.02 | (0.769) | -0.05 | (0.248) | 0.12 | (0.036) | -0.01 | (0.878) | -0.01 | (0.939) |
| Crude at Second Follow-Up | -0.07 | (0.484) | 0.09 | (0.443) | 0.05 | (0.775) | -0.01 | (0.967) | -0.01 | (0.147) |
| Adjusted at Second Follow-up | -0.10 | (0.316) | 0.05 | (0.540) | 0.02 | (0.898) | 0.08 | (0.473) | 0.08 | (0.243) |
