# Supplementary material for: Outcomes of a social media campaign to promote COVID-19 vaccination in Nigeria
Source: PLoS One. 2023 Sep 15;18(9):e0290757. doi: 10.1371/journal.pone.0290757 (PMC10503765; doi:10.1371/journal.pone.0290757)
Supplement: S2 File — (DOCX) [file pone.0290757.s002.docx]

**CODEBOOK for PLOS One Dataset**

| VARIABLE NAME | DEFINITION |
| --- | --- |
| userid | Auto-generated variable |
| agegrp0 | How old are you?  1. 18-29 years  2. 30-39 years  3. 40-49 years  4. 50-59 years  5. 60+ years |
| benefit0  benefit1  benefit2 | When I think about getting vaccinated against COVID-19, I weigh the benefits and risks to make the best decision possible.  1. Strongly disagree  2. Disagree  3. Neither agree nor disagree  4. Agree  5. Strongly agree |
| close0  close1  close2 | Of the people close to you, what proportion of them would want you to get the COVID-19 vaccine?  1. A few (1-20%)  2. Some (21-40%)  3. Many (41-60%)  4. Most (61-80%)  5. Nearly all or all (81-100%) |
| edu0 | What is the highest educational level or degree that you have attained?  1. Primary school  2. Secondary school  3. Diploma  4. Bachelors  5. Masters  6. PhD  7. Other |
| empsect0 | Do you work in one of these health care roles?  1. Not a health care worker  2. Medical doctor  3. Nurse/midwife  4. Laboratory staff  5. Pharmacist  6. PPMV/chemists  7. Community health worker  8. Other public health practitioner |
| everyone0  everyone1  everyone2 | When everyone else is vaccinated against COVID-19, I don’t have to get vaccinated too.  1. Strongly disagree  2. Disagree  3. Neither agree nor disagree  4. Agree  5. Strongly agree |
| family0  family1  family2 | Your family members think it is important for everyone to get a COVID-19 vaccine.  1. Strongly disagree  2. Disagree  3. Neither agree nor disagree  4. Agree  5. Strongly agree |
| fivec0*  fivec1*  fivec2* | Row mean of benefit, everyone, safe (reversed), stress, unnecessary |
| friends0  friends1  friends2 | Your friends think it is important for everyone to get a COVID-19 vaccine.  1. Strongly disagree  2. Disagree  3. Neither agree nor disagree  4. Agree  5. Strongly agree |
| gender0 | What is your gender?  1. Man  2. Woman  3. Prefer not to say |
| healthc0  healthc1  healthc2 | How many people who work in health care in Nigeria do you think will get the COVID-19 vaccine when it becomes available?  1. A few (1-20%)  2. Some (21-40%)  3. Many (41-60%)  4. Most (61-80%)  5. Nearly all or all (81-100%) |
| ltfu1* | 0. Not lost before follow-up 1  1. Lost before follow-up 1 |
| ltfu2* | 0. Not lost before follow-up 2  1. Lost before follow-up 2 |
| nigerian0  nigerian1  nigerian2 | How many people in Nigeria do you think will get the COVID-19 vaccine when it becomes available?  1. A few (1-20%)  2. Some (21-40%)  3. Many (41-60%)  4. Most (61-80%)  5. Nearly all or all (81-100%) |
| norms0*  norms1*  norms2* | Row mean of close, family, friends, healthc, and nigerian |
| religion0 | What is your religion?  1. Catholic  2. Islam  3. Other  4. Other Christian  5. Traditionalist |
| safe0  safe1  safe2 | I am confident that COVID-19 vaccines are safe and effective.  1. Strongly disagree  2. Disagree  3. Neither agree nor disagree  4. Agree  5. Strongly agree |
| stratum_location0 | State names as provided by Facebook |
| stress0  stress1  stress2 | Everyday stress prevents me from getting a COVID-19 vaccine.  1. Strongly disagree  2. Disagree  3. Neither agree nor disagree  4. Agree  5. Strongly agree |
| treat* | Derived from stratum_location  0. Comparison state  1. Treatment / campaign state |
| unneces0  unneces1  unneces2 | Vaccination against COVID-19 is unnecessary.  1. Strongly disagree  2. Disagree  3. Neither agree nor disagree  4. Agree  5. Strongly agree |
| vaxxed0*  vaxxed1*  vaxxed2* | Derived from vaccinated_already  0. Not vaccinated (4)  1. Vaccinated (1, 2, or 3) |
| vaccinated_already0  vaccinated_already1  vaccinated_already2 | Have you received a COVID-19 vaccine?  1. Yes, a single dose vaccine  2. Yes, the first dose of a two-dose regimen  3. Yes, both doses of a two-dose regimen  4. No |

NOTES: A 0, 1, or 2 at the end of a variable name indicates baseline, first follow-up, and second follow-up. Asterisks (*) indicated derived variables; all other variables are original.
